# Disability-adjusted-life-years losses in postmenopausal women with osteoporosis: a burden of illness study

**DOI:** 10.1186/s12889-015-1684-7

**Published:** 2015-04-02

**Authors:** Josep Darbà, Lisette Kaskens, Nuria Pérez-Álvarez, Santiago Palacios, José Luis Neyro, Javier Rejas

**Affiliations:** Department of Economics, Universitat de Barcelona, Diagonal 690, 08034 Barcelona, Spain; BCN Health Economics & Outcomes Research S.L., Travessera de Gracia 62, 5-6, 08006 Barcelona, Spain; Instituto Palacios de Salud y Medicina de la Mujer, Calle Antonio Acuña, 9, 28009 Madrid, Spain; Department of Obstetrics and Gynaecology, Hospital Universitario Cruces, Gran Vía 81-4, 48011 Bilbao, Spain; Health Economics and Outcomes Research Department, Pfizer, S.L.U., Avda. Europa 20B. Parque Empresarial la Moraleja, 28108 Alcobendas, Madrid Spain

**Keywords:** Disability-adjusted life year, Burden of illness, Osteoporosis, Menopause, Disability weights, Drug-based therapy, Osteoporotic risk fracture factors

## Abstract

**Background:**

To estimate the disability-adjusted life years (DALY) in a nationwide representative sample of postmenopausal women with osteoporosis. The effects of drug-based therapy and risk factors for osteoporotic bone fractures on DALY losses were also explored.

**Methods:**

DALY were estimated based on participant’s clinical characteristics and Health-Related Quality-of-Life (HRQoL) data obtained from a cross-sectional, epidemiological one-visit study (the GINERISK study). The study enrolled postmenopausal women (at least 12-months after their last menstrual period) with osteoporosis, above 18-years old, who attended Spanish outpatient Gynaecology clinics. HRQoL was assessed using the generic SF-12v2 questionnaire, which was used to derive disutility values. Mortality rates were extracted from the Spanish national statistics database. Factors explored to be associated with DALY losses were examined using ANOVA, ANCOVA and MANCOVA models.

**Results:**

DALY could be computed in 2,782 (67%) out of 4,157 postmenopausal women, with a mean (95% CI) age of 61.0 (60.7-61.2) years. Overall individual undiscounted DALY per woman were 6.1 (5.9-6.2), resulting to be significantly higher in women with severe osteoporosis with prior bone fracture; 7.8 (7.2-8.4) compared to osteoporotic women [5.8 (5.6-6.0)] or postmenopausal women with a BMD > −2.5 T-score that received a drug-based therapy [6.2 (5.8-6.5)]; F = 27.0 (*P* < 0.01). Models explaining the variation in the levels of health based on the use of a selective estrogen receptor modulator (SERM) or possession of risk factors for osteoporotic BF were found (*P* < 0.05).

**Conclusions:**

DALY losses were considerable amongst postmenopausal women with osteoporosis. Not having a prior bone fracture, being older, using a SERM and having less osteoporotic risk factors were all linked to less DALY losses.

## Background

Osteoporosis is a major public health problem characterized by a loss of bone mineral density (BMD) associated with fragility fractures [1-3]. In developed countries osteoporosis-associated fractures form one relevant cause of mortality and a major cause of disability for post-menopausal women [4,5]. Patients having osteoporosis that experienced bone fractures showed to have a decrease in health related quality of life (HRQoL) due to pain, deformity and/or disability, as well as death [6].

Therefore it is important to focus attention on the identification of women with a high risk of fragility fracture, apart from the identification of patients with osteoporosis, which are usually diagnosed exclusively by BMD according to WHO criteria. These four general diagnostic categories for women [7-9] expressed in relation to a reference population in standard deviation (SD) units proposed include: normal, with a BMD of less than 1 SD below the reference population (T-score > −1); osteopenia, with a BMD of more than 1 SD below the reference population but less than 2.5 SD (T-score ≤ −1 and > −2.5); osteoporosis, with a BMD of 2.5 SD or higher (T-score ≤ −2.5) below the reference population; severe osteoporosis with a BMD value of 2.5 SD or higher below the mean of the reference population in the presence of one or more fragility fractures.

Although BMD is an important component of bone fracture risk, several other risk factors have also been demonstrated to affect the risk of bone fractures. These risk factors include but are not limited to age, a prior fragility fracture, a parental history of hip fracture, smoking, the use of systemic corticosteroids, excess alcohol use, rheumatoid arthritis, low body weight, etc., [10]. The independent contribution of each of these risk factors can be integrated to estimate fracture probability with or without the use of BMD and should be taken into account when performing a global risk evaluation [11].

At a global level several studies have quantified the burden of osteoporosis [12-14] which is based on the most important consequences of osteoporosis, in specific on fragility fractures. For this reason, fractures are a measure of the disease burden of osteoporosis. But the problem is that this measure is imprecise as fractures are a specific state in themselves which are associated with further burdensome consequences like losses of functioning and fear [15]. Therefore information on disability and fatal consequences as life-years lost due to premature mortality are more important information which is combined in disability-adjusted life years (DALY). Premature mortality entails estimating the average time a person would have lived had he or she not died prematurely [16]. This estimation inherently incorporates age and death, rather than merely the occurrence of death itself [17]. To date, individual DALY losses due to osteoporosis for Spanish postmenopausal women have not been estimated.

The aim of this study was to estimate individual DALY losses based on participants’ clinical characteristics and HRQoL data collected in a cross-sectional, epidemiological one-visit study, called the GINERISK study [18], among a nationwide representative sample of postmenopausal women with osteoporosis. As described earlier several factors have been demonstrated to affect bone fracture risk [5], and therefore may affect health levels of osteoporotic women. Therefore the relationship between drug-based therapy and risk factors for osteoporotic bone fracture was also explored, to determine its association with improvements in the health levels of these postmenopausal osteoporotic women, in terms of more or less DALY losses.

## Methods

### Study sample and methods of sampling

Participant data used for the estimation of DALY losses was extracted from the GINERISK study [18]. In brief, this was a nationwide study carried out during 2011 at outpatient clinics of Gynaecology in Spain. Postmenopausal women above 18 years of age attending these clinics were invited to participate in the study. Inclusion criteria for the study were being a postmenopausal woman (last menstruation over more than 12 months, either natural menopause or surgically- or drug-induced menopause) with a diagnosis of osteoporosis (spinal BMD T-score < −2,5 according to the WHO criteria and identified by Bone Densitometry - DXA) within two years prior to the study visit. All participants had to sign an informed consent form.

Among the women who underwent a DXA during the study visit, around 20% were diagnosed to be non-osteoporotic according to the WHO criteria. However, these women had been diagnosed with osteoporosis within the last 2 years (according to the inclusion criteria in the study) and received treatment under supervision of their gynecologist for their condition, which subsequently lead to reversal of their condition at the time of the Ginerisk one-visit study. Participants were excluded if they received treatment with hormones or any other psychopharmacologic agent, or if they were not able to understand patient-reported-outcomes questionnaires written in Spanish for any reasons.

A stratified multistage probability sample without replacement was drawn. The sampling frame included all health regions of the 17 Autonomous Communities within Spain. The first stage consisted of the selection of Gynecology clinics within each health region. The number of selected clinics in each region was proportional to the population density of the specific region. In the second stage, one gynecologist per clinic was chosen at random among those who had previously participated in clinical and/or epidemiological research in the field of gynecology, and invited to participate. The third stage consisted of participant selection with a systematic sampling strategy from the daily list of all participants that had an appointment with each of the participating gynecologists and that had met the previously mentioned inclusion criteria.

During one single visit the gynaecologists completed the individual case report forms (CRFs) with information collected from a questionnaire administered by the same gynaecologist. Supplementary data obtained from participant’s records on socio-demographics, clinical characteristics, medication utilization, and HRQoL was also collected. Medication utilization related to osteoporosis was collected from the patients’ medical record and included the following type of therapies to treat osteoporosis: calcium supplement, calcium + vitamin D, bisphosphonates, selective estrogen receptor modulators (SERM) or other drugs. Information on regular physical exercise was asked directly to the participant. In addition to the required initial diagnose of osteoporosis using a DXA, BMD was also measured during the study visit using a DXA. According to the current values obtained by DXA (T-score) and the WHO criteria, all women were re-classified into three groups: osteoporosis (T-score ≤ −2.5), severe osteoporosis (T-score ≤ −2.5 in the presence of one or more prior bone fractures) and women with a T-score > −2.5. The study was conducted in accordance with the ethical principles of the Declaration of Helsinki and was approved by the Ethical Committee of the Hospital de la Princesa in Madrid.

### Sample size

The sample size was established following the guidelines of the International Conference on Harmonization [18], in order to be able to draw firm conclusions in relation to the established study objectives in the original, or GINERISK study and no adjustments were carried out for the analysis included here. Nevertheless, calculations were carried out posteriori to recalculate the sample power. This recalculation showed that the current study had a minimum power of 90% with a confidence level of 95% to detect a mean difference of 1.0 (SD: 2.7) in DALY losses between the groups with the smallest sample of 150 participants per group as a minimum. The study sample of the Ginerisk study included a total of 4,157 participants, but for the current analysis 1,375 participants were excluded as information on BMD, date of diagnosis of osteoporosis or on HRQoL were not available. The final population for DALY estimation consisted of 2,782 (67%) Spanish postmenopausal women with a diagnosis of osteoporosis (spinal BMD T-score < −2,5 according to WHO criteria and identified by DXA) within the two years prior to the study visit with information on BMD, with a date of diagnosis of osteoporosis and available HRQoL values at the time of data collection. In order to be sure that missing data could not be allocated to a special group of participants, characteristics of the participants with a DALY value were compared univariantly with participants without a DALY value by means of chi-square or t-test. No significant differences between both groups were observed in age, smoking and BMI categorized by 20 kg/m^2^.

### Disability-adjusted life-years

A detailed description on the methodology used for DALY estimation has been described by Fox-Rushby & Hanson [19]. DALY as a measure of the level of health is the sum of the number of years of life lost (YLL) due to premature mortality plus the number of years lived with a disability (YLD). In this study undiscounted and discounted DALY were estimated, using a 3% discount without applying any age weighting factor [20,21].

### Years of life lost

YLL is an estimation of the number of years lost as a result of premature death based on a predetermined life expectancy. Future YLL were computed for each individual participant by calculating the expected individual life expectancy of each woman and the age of death due to osteoporosis or severe osteoporosis. As no mortality of women with osteoporosis or severe osteoporosis was actually observed in the Ginerisk study, the estimation of YLL concerns an estimation of the possible premature death of the study participants due to their condition. For the estimation of future life years lost, the standard life expectancy (i.e. 84.6 years for Spanish women) from a given time until the age of death and mortality data associated with osteoporosis and severe osteoporosis were obtained from the Spanish National Statistics Institute [22]. It was assumed that women with a T-score > −2.5, had a standard life expectancy similar to the general population. Therefore YLL were only estimated for osteoporotic and severe osteoporotic women who showed excess mortality due to osteoporosis. Excess mortality associated with osteoporosis was observed for severe osteoporotic women aged 70 years or more and showed to increase with age. The excess mortality rates applied respectively were: 8/100.000 for women aged 70–74, 111/100.000 women aged 75–79, 363/100.000 women aged 80–84 and 1.716/100.000 for women aged over 85. Excess mortality for osteoporotic women was lower that than for severe osteoporotic women. The excess mortality rates applied respectively were: 1/100.000 for women aged 60–69, 2/100.000 women aged 75–79, 9/100.000 women aged 80–84 and 40/100.000 for women aged over 85.

For example, a woman with severe osteoporosis, with an age falling in the category 75–79 years old, had a mortality rate of 2 per 100.000 associated with osteoporosis. Based on an average age of 77.5, the interpolated life expectancy of an osteoporotic woman was estimated to be 11.61 years which is equal to the life years women in this age category would lose. The losses of 11.61 years per 2 out of 100,000 women in the age category 75–79 years old would lead to ((2*11.61)/100,000) approximately 0,0002 undiscounted life years lost due to severe osteoporosis for a women in this age category. For several age categories including osteoporotic and severe osteoporotic women no mortality rate was reported as no mortality due to osteoporosis was observed in the data from the Spanish National Statistics Institute [22].

### Years of life lived with disability

YLD is an estimate of the number of years that a participant lives with disability. YLD were estimated for each individual postmenopausal woman based on the loss in HRQoL associated with osteoporosis and the expected individual life span at the age of diagnosis. Data on the age of diagnosis of osteoporosis and HRQoL to compute disability weights were derived from the GINERISK study.

HRQoL data used to compute disability weights was measured using the generic version 2 of the Medical Outcomes Study (MOS)–Short Form (SF) with a 12 item questionnaire (SF-12v2). The SF-12 survey generates a physical component summary (PCS) and mental component summary (MCS). Utility regression algorithms and coefficients by Franks et al., [23] provided inputs for the conversion of PCS and MCS scores of the SF-12 in EuroQol EQ-5D interval-level scores anchored at 0 (death) and 1 (perfect health) that represent preferences for particular health states. Mean PCS and MCS scores were used to calculate a utility value for each individual resulting respectively in 43.09 and 47.57, and were adjusted to the mean health at the same age of the population considered in the current study. Individual disability weights, based on the predicted utility values, were calculated according to the following formula of D = 1 – Q [24], in which D is the disability weight and Q is the quality of life weight. The disability weight ranges from 0 to 1, in which 0 = perfect health and 1 = death.

For example YLD for a woman with an age falling in the category 75–79 years old, were based on the number of years that the women has lived with disability due to her condition. A woman with an average age of 77.5 and a time since diagnosis of 4 years was supposed to have lived with her condition based on an interpolated life expectancy of 11.61 at the age of 77.5, for approximately 15.61 years. Her undiscounted life years lived with disability would result in 15.61 years corrected for a disability weight based on her individual HRQoL loss. If this patient would have had a disability weight of 0.5, then, she would have an YLD value of 7.805.

### Statistical analysis

A descriptive analysis on variables including socio-demographics, clinical characteristics, personal background and osteoporosis therapy recorded in the Ginerisk study was conducted, reporting absolute and relative frequencies for qualitative variables and main centralization and dispersion measures for quantitative variables. To compare the independent samples, Pearson’s chi-square test (χ2) (or Fisher’s exact test for 2 × 2 contingency tables or likelihood ratio for mxn contingency tables, if necessary) was used for qualitative variables, and the Student’s T test, one-way analysis of variance (ANOVA) or its non-parametric equivalents (Mann–Whitney U test or Kruskal-Wallis H test) were used for quantitative variables. The statistical significance level was established at *P* < 0.05.

DALY were analyzed with descriptive statistics, using univariate ANOVA tables. Subsequently, paired-comparison analyses were completed for factors showing significant group differences in univariate ANOVA tables. A post-hoc Games-Howell test, was used to evaluate specific group contrasts and to correct for unequal variances to give an overall significance level of alpha = 0.05. All values were presented as a mean and a 95% confidence interval.

To find out if health levels or DALY losses varied by their association with risk factors and the use of a drug based therapy, univariate and multivariate regression analysis were carried out on the whole study sample. The impact of risk factors on health levels was assessed using an ANCOVA and MANCOVA model with BMD, prior bone fracture, BMI < 20 kg/m^2^, active smoking, alcohol consumption (equal or above 30 g/day), family antecedents of osteoporosis or/and hip fracture, rheumatoid arthritis and receiving a corticosteroids-based therapy as covariates. The impact of using a drug-based therapy with a SERM was assessed using an ANCOVA model with age, BMD, prior osteoporotic bone fractures, number of risk factors for osteoporotic bone fracture, exercise and ongoing treatment with calcium or calcium + vitamin D as covariates. Data was analyzed with SPSS® version 18.0 for Windows (SPSS; Chicago, IL).

## Results

### Descriptive results

In total 4,157 women participated in the Ginerisk study of which 2,782 were postmenopausal women that had a value for BMD (T-score) and a HRQoL score. Table 1 provides data on socio-demographics, clinical characteristics, background with co-morbidities and osteoporosis, for the three groups categorized according to their BMD value into severe osteoporosis (n = 272), osteoporosis (n = 1,958) and postmenopausal women with a current T-score > −2.5 (n = 552). The mean age of the whole study population was 61.15 (95% CI 60.90-61.40) years.

**Table 1:** **Socio-demographics, clinical characteristics and participant background by study group**

**Variable**	**Study group by BMD**			
	**Severe osteoporosis with prior BF (n = 272)**	**Osteoporosis (n = 1,958)**	**T-score > −2.5 (n = 552)**	**F or Chi-** ^**2**^ **(p value)** ^**₤**^
*Socio-demographic data*				
Age (years)	63.3 ± 7.3	60.9 ± 7.4	59.9 ± 6.6	20.3 (p < 0.001)
Age group (%)				
≤44	0.0	0.5	0.2	38.4 (p < 0.001)
45-49	1.5	3.7	4.2	
50-54	9.6	13.6	16.8	
55-59	21.7	28.4	31.0	
60-64	28.7	24.9	25.0	
≥65	38.6	28.9	22.8	
Employment situation (%)				
Working	28.3	41.3	45.1	37.4 (p < 0.001)
Transitory sick leave	4.0	2.2	1.6	
Permanent disability	1.5	0.7	0.5	
Unemployment	3.3	3.6	2.0	
Retired	29.0	20.1	16.7	
Housewife	33.8	32.1	34.1	
Education level (%)				
None	12.2	6.5	5.3	23.7 (p = 0.003)
Primary	39.6	34.4	36.6	
Secondary	25.2	29.3	28.8	
Undergraduate	13.3	16.7	14.5	
Degree	9.6	13.2	14.9	
Environment (%)				
Rural	16.6	9.9	11.6	0.1 (p = 0.702)
Semi-urban	21.8	22.7	20.1	
Urban	34.3	38.8	45.0	
Metropolitan	27.3	28.6	23.2	
*Clinical characteristics*				
BMI (kg/m^2^)	25.8 ± 4.1	25.6 ± 4.3	25.7 ± 4.4	0.4 (p = 0.675)
Cigarettes/day	1.6 ± 0.7	1.6 ± 0.7	1.8 ± 0.7	2.2 (p = 0.113)
Smoking (%)				
Non-smoker	67.3	67.7	66.7	1.1 (p = 0.889)
Former smoker	16.2	17.8	18.2	
Smoker	16.5	14.6	15.1	
Alcohol consumption, any (%)	22.2	17.5	18.7	3.7 (p = 0.156)
Alcohol consumption > 30 gr/day (%)	1.9	0.8	1.5	4.2 (p = 0.125)
*Background of co-morbidities(%)*				
Diabetes mellitus				
No	81.0	93.2	94.5	51.9 (p < 0.001)
Type I	3.7	1.2	2.6	
Type II	15.3	5.6	2.9	
Hypertension (%)	37.8	21.9	22.7	33.2 (p < 0.001)
Rheumatoid arthritis	7.6	4.3	3.7	6.9 (p = 0.031)
Anorexia nervosa	0.4	0.3	0.4	0.1 (p = 0.968)
Hyperparathyroidism	0.8	0.3	0.4	1.0 (p = 0.602)
Hyperthyroidism	3.8	2.5	1.3	5.1 (p = 0.078)
Chronic liver diseases	0.8	0.7	0.4	0.8 (p = 0.664)
Malabsorption syndrome	1.9	0.2	0.6	10.0 (p = 0.007)
*Osteoporosis data*				
Age at diagnosis (years)	59.9 ± 6.8	58.9 ± 6.9	58 ± 6.4	8.7 (p < 0.001)
BMD (DXA)	−2.9 ± 0.4	−2.8 ± 0.5	−0.9 ± 1.9	990.3 (p < 0.001)
# of risk factors for osteoporotic BF	1.9 ± 0.8	0.5 ± 0.7	0.5 ± 0.7	495.7 (p < 0.001)
# of risk factors for osteoporosis	6.9 ± 0.3	5.0 ± 1.8	4.9 ± 1.8	172.9 (p < 0.001)
Time from diagnosis (years)	3.4 ± 3.8	2.1 ± 2.8	2.1 ± 2.8	24.0 (p < 0.001)

Note: Values are mean (standard deviation) or percentage relative to total in the group. ^₤^Chi^2^ may be lineal for trend or likelihood ratio. Environment; rural (≤10,000 inhabitants), semi-urban (>10,000 to ≤30,000 inhabitants), urban (>30,000 to ≤200,000 inhabitants) and metropolitan (>200,000 inhabitants). BF = Bone fracture.

Significant differences were observed between the three groups in age (*P* < 0.001), employment situation (*P* < 0.001) and education level (*P* = 0.002) with the exception of environment (*P* = 0.973). The presence of co-morbidities including type 1 and type 2 diabetes mellitus, hypertension, rheumatoid arthritis, hyperthyroidism, and malabsorption syndrome showed to be significantly different for the three groups (*P* ≤ 0.01). Significant differences (*P* < 0.001) between the three groups were also found for the mean age of diagnosis of osteoporosis, BMD, number of risk factors for osteoporotic bone fractures, osteoporosis itself and time passed since diagnosis.

Table 2 provides the data on treatment for osteoporosis for all three groups which consisted of hygiene and health measures, calcium supplements, calcium + vitamin D, exercise, and drug therapy, the latter comprising bisphosphonates, SERM, and other drugs. The data showed that most postmenopausal women independent of its BMD value used some kind of treatment although no significant differences existed between the different therapies used. Bisphosphonates and other drug treatments were more frequently used. Significant differences exists in the use bisphosphonates among women with severe osteoporosis, while a SERM was significantly more used in women with osteoporosis or postmenopausal women with a T-score > −2.5.

**Table 2:** **Osteoporosis therapy background by study group**

**Variable**	**Study group by BMD**			
	**Severe osteoporosis with prior BF (n = 272)**	**Osteoporosis (n = 1958)**	**T-score > −2.5 (n = 552)**	**F or Chi-** ^**2**^ **(p value)** ^**₤**^
*Osteoporosis therapy (%)*				
Hygiene/health measures	90.9	91.4	89.6	1.4 (p = 0.489)
Calcium supplement	12.2	10.4	14.5	5.6 (p = 0.062)
Calcium + vitamin D	80.0	81.9	72.7	16.8 (p < 0.001)
Exercise	45.2	42.3	42.3	0.7 (p = 0.694)
Biphosphonates	61.7	49.0	43.5	19.1 (p < 0.001)
SERM	26.5	42.3	47.1	26.3 (p < 0.001)
Other drug therapy	12.2	7.0	5.5	10.0 (p = 0.007)

Note: Values are percentage relative to total in the group. ^₤^Chi^2^ may be lineal for trend or likelihood ratio.

### Individual DALY

DALYs were computed for 2,782 postmenopausal women, for whom an age of diagnosis of osteoporosis, BMD (T-score) value and HRQoL score were available. In terms of the level of health, individual DALY losses undiscounted and discounted were expressed in mean (95% CI) for the total study population, and by group according to BMD and age as shown in Table 2. Total undiscounted DALY losses for the group with severe osteoporosis (Table 3) were significantly higher with 7.8 (95% CI; 7.2-8.4) than for the group with osteoporosis and the group with a T-score > −2.5 with respectively 5.8 (95% CI; 5.8-6.5) and 6.2 (95% CI; 5.8-6.5) (*P* < 0.001). Similar results were observed when future DALY losses were discounted with 3%.

**Table 3:** **DALY losses in mean (CI95%), undiscounted and discounted by BMD and age**

**Individual DALYs undiscounted**	**All ages**		**Women < 65 years of age**		**Women ≥ 65 years of age**	
	**Mean**	**(95% CI)**	**Mean**	**(95% CI)**	**Mean**	**(95% CI)**
Severe osteoporosis with prior BF (n = 272)	7.8^a,**b**^	(7.2 8.4)	9.0^a,**b**^	(8.2, 9.8)	5.9^d^	(5.3, 6.6)
Osteoporosis (n = 1,958)	5.8^**c**^	(5.6, 6.0)	6.1^**c**^	(5.8, 6.4)	4.9^e^	(4.6, 5.2)
T-score > −2.5 (n = 552)	6.2^**c**^	(5.8, 6.2)	6.5^**c**^	(6.0, 6.9)	5.0	(4.6, 5.5)
Total study population (n = 2,788)	6.1	(5.9, 6.2)	6.4	(6.2, 6.7)	5.1	(4.9, 5.3)
**Individual DALYs discounted**	**All ages**		**Women < 65 years**		**Women ≥ 65 years of age**	
	**Mean**	**(95% CI)**	**Mean**	**(95% CI)**	**Mean**	**(95% CI)**
Severe osteoporosis with prior BF (n = 272)	5.5^a,**b**^	(5.1, 5.9)	6.1^a,**b**^	(5.5, 6.6)	4.6^d,f^	(4.2, 5.1)
Osteoporosis (n = 1,958)	4.0^**c**^	(3.9, 4.1)	4.1^**c**^	(3.9, 4.2	3.8^e^	(3.6, 4.0)
T-score > −2.5 (n = 552)	4.2^**c**^	(4.0, 4.4)	4.3^**c**^	(4.0, 4.6)	3.9^g^	(3.5, 4.3)
Total study population (n = 2,788)	4.2	(4.1, 4.3)	4.3	(4.2, 4.4)	3.9	(3.8, 4.1)

Note: ^a^ *P* < 0.001 versus osteoporotics; ^b^ *P* < 0.001 versus postmenopausal women T > −2.5; ^c^ *P* < 0.001 versus severe osteoporosis with prior BF; ^d^ *P* = 0.01 versus osteoporotics; ^e^ *P* = 0.01 versus severe osteoporosis with prior BF; ^f^ *P* < 0.05 postmenopausal women T > −2.5; ^g^ *P* < 0.05 versus severe osteoporotics with prior BF.

DALY losses in postmenopausal women <65 years of age undiscounted and discounted were higher than for postmenopausal women ≥ 65 years of age for all three study groups (Table 2). DALY losses were significantly higher for the group with severe osteoporosis aged <65 years compared to the group with osteoporosis and the group with a T-score > −2.5. For postmenopausal women ≥ 65 years of age a significantly higher losses in undiscounted and discounted DALY was observed for the severe osteoporotic group compared to with the group with osteoporosis.

Only a significant difference in discounted DALY losses was observed between the severe osteoporotic group and the group with a T-score > −2.5.

### Variables associated with improved levels of health

The results of the covariate and multivariate regression analysis to determine the impact of risk factors on DALY losses in the total study population are represented in Table 4. The results showed that not having a prior osteoporotic bone fracture lead to significantly lower undiscounted DALY losses (Constant = −2.0), as well as less alcohol consumption than 30 g/day (Constant = −2.4), not having rheumatoid arthritis (Constant = −2.9), no prior family antecedents of osteoporosis (Constant = −0.4), and not using corticosteroids (Constant = −3.6). Similar associations were observed for undiscounted and discounted DALY losses with the exception of the impact of family antecedents of osteoporosis. Table 4 also shows the results of the MANCOVA analysis by adding all risk factors to the model at the same time. Having a higher BMD value, a prior osteoporotic bone fracture, rheumatoid arthritis, family antecedents of osteoporosis and the use of corticosteroids showed to significantly affect DALY losses. A similar model was found for discounted DALY losses, with the exception of the impact of family antecedents of osteoporosis.

**Table 4:** **Variables associated with DALY losses adjusted by risk factors**

Variables*	**ANCOVA** ^**a**^			**MANCOVA** ^**b**^			**Undiscounted DALYs**		
	**Constant**	**(95% CI)**	***P*** **-value**	**Constant**	**(95% CI)**	***P*** **-value**	**Mean**	**(95% CI)**	***P*** **-value**
BMD	0.1	(0.0, 0.3)	0.066	0.2	(0.1, 0.3)	0.004	-	-	-
Prior osteoporosis BF (No)	−2.0	(−2.5, −1.4)	<0.001	−1.8	(−2.3, −1.2)	<0.001	-	-	-
BMI < 20 kg/m2 (No)	0.0	(−0.7, 0.8)	0.898	-	-	-	6.1	(5.2, 6.9)	-
Smoking status (Non-smoker)	−0.2	(−0.7, 0.2)	0.336	-	-	-	6.0	(5.9, 6.2)	-
Alcohol consumption ≥30 g/day (No)	−2.4	(−4.0, −0.8)	0.003	-	-	-	6.2	(5.9, 6.0)	*P* < 0.01
Rheumatoid arthritis (No)	−2.9	(−3.6, −2.1)	<0.001	−2.8	(−3.5, −2.0)	<0.001	5.9	(5.8, 6.1)	*P* < 0.01
Family antecedents of osteoporosis (No)	−0.4	(−0.7, 0.0)	0.033	−0.4	(−0.8, −0.1)	0.016	5.8	(5.6, 6.0)	*P* < 0.05
Use of corticosteroids (No)	−3.6	(−5.9, −1.4)	0.002	−3.6	(−5.9, −1.2)	0.003	6.0	(5.9, 6.2)	*P* < 0.01
**Variables**	**ANCOVA** ^**a**^			**MANCOVA** ^**b**^			**Undiscounted DALYs**		
	**Constant**	**(95% CI)**	***P*** **-value**	**Constant**	**(95% CI)**	***P*** **-value**	**Mean**	**(95% CI)**	***P*** **-value**
BMD	0.1	(0.0, 0.2)	0.102	0.1	(0.0, 0.2)	0.004	-	-	-
Prior osteoporosis BF (No)	−1.5	(−1.8, −1.1)	<0.001	−1.4	(−1.8, −1.1)	<0.001	-	-	-
BMI < 20 kg/m2 (No)	0.2	(−0.3, 0.7)	0.462	-	-	-	4.1	3.5, 4.6	-
Smoking status (Non-smoker)	0.0	(−0.3, 0.3)	0.793	-	-	-	4.2	4.1, 4.3	-
Alcohol consumption ≥30 g/day (No)	−1.5	(−2.5, −0.4)	0.008	-	-	-	4.2	4.1, 4.3	*P* < 0.01
Rheumatoid arthritis (No)	−2.2	(−2.7, −1.6)	<0.001	−2.1	(−2.6, −1.6)	<0.001	6.4	5.8, 4.2	*P* < 0.01
Family antecedents of osteoporosis (No)	−0.2	(−0.4, 0.1)	0.132	-	-	-	4.0	3.9, 4.7	-
Use of corticosteroids (No)	−2.3	(−3.9, −0.8)	0.002	−2.2	(−3.7, −0.6)	0.007	4.2	4.1, 4.3	*P* < 0.01

Notes: ^a^Model build for each factor adjusted by BMD and prior osteoporotic BF; ^b^Model calculated with all the factors at the same time; NS = Not significant. The global mean (95% CI) for the undiscounted DALYs multivariate model was 14.6 (12.2; 17.1) and 10.0 (8.3; 11.6) for the discounted DALYs. *For categorical variables the reference category is given between brackets.

Table 5 shows the results of the impact of a drug-based therapy with SERM on DALY losses for the total study population using a covariate regression analysis. Not using a SERM showed to increase DALY losses (Constant = 0.6) as well as having risk factors for osteoporosis (Constant = 0.3). Being younger (Constant = −0.1) and not having a prior osteoporotic bone fracture (Constant = −1.6) showed to reduce DALY losses. Similar associations with the use of a SERM were observed for discounted DALY losses.

**Table 5:** **Variables associated with DALY losses adjusted by drug based therapy**

**Undiscounted DALYs**	**ANCOVA**		
Variables*	**Constant**	**95% CI**	***P*** **-value**
BMD	0.1	(−0.3, 0.5)	0.607
Age	−0.1	(−0.1, −0.1)	<0.001
Prior osteoporotic BF (No)	−1.6	(−2.3, −0.9)	<0.001
Prior treatment with calcium (No)	−0.6	(−0.7, 0.2)	0.121
Prior treatment with calcium + vitamin D (No)	−0.1	(−0.7, 0.6)	0.877
Exercise (No)	0.3	(−0.1, 0.7)	0.188
Risk factors for osteoporosis	0.3	(0.1, 0.6)	0.018
Use of SERM (No)	0,6	(0.1, 1.0)	0.010
**Discounted DALYs**	**ANCOVA**		
Variables*	**Constant**	**95% CI**	***P*** **-value**
BMD	0.1	(−0.2, 0.3)	0.567
Age	0.0	0.0	0.003
Prior osteoporotic BF (No)	−1.1	(−1.6, −0.6)	<0.001
Prior treatment with calcium (No)	−0.5	(−1.0, 0.1)	0.090
Prior treatment with calcium + vitamin D (No)	−0.1	(−0.5, 0.4)	0.831
Exercise (No)	0.2	(−0.1, 0.5)	0.189
Risk factors for osteoporosis	0.2	(0.3, 0.4)	0.019
Use of SERM (No)	0.4	(0.1, 0.7)	0.010

*For categorical variables the reference category is given between brackets.

## Discussion

The most innovative part of this study is the use of individual DALY losses to measure the level of health in postmenopausal women according to their BMD value from the data collected by the GINERISK study. The collection of generic HRQoL allowed us to compute disability weights for the estimation of individual YLD due to osteoporosis. Generic HRQoL showed to be worse in severe osteoporotic participants compared with the rest of the study population, in relation to the Spanish general female population [18]. Accordingly, as observed in this study, YLD were higher for severe osteoporotic participants than for osteoporotic participants and postmenopausal participants with a T-score > −2.5 resulting in higher DALY losses which is in line with literature [5,25,26] . Although no specific information on the type of prior bone fractures was known for participants with severe osteoporosis in the Ginerisk study, data from literature [27] showed that mortality risk increases and remains higher than the age-matched general population mortality rate for several years following hip and vertebral fractures which explains why premature death or YLL in severe osteoporotic participants is higher than in the other groups.

DALY losses in this study were presented undiscounted and 3% discounted without applying an age weighting factor according to the methodology described by Fox-Rushby & Hanson [19]. The 3% discount rate is similar to the rate applied in the World Bank and Global Burden of Disease project [28,29]. No age-weighting was applied to give DALY losses different values at different ages which imply that the relative value of a year of life rises rapidly from birth to a peak in the early twenties, after which it declines steadily [30]. This might be a limitation of the study but on the other hand applying age-weights has also been criticised and evaluated as unacceptable on equity grounds [31], not empirically based [32] and adding complexity to burden of disease analyses that obscures the method and makes little overall difference to the rankings of diseases and injuries.

In this study, DALY losses were shown to be affected by risk factors for osteoporosis or the use of a drug based treatment with SERM. An explanation for the association between DALY and risk factors or using a drug-based treatment with SERM is that both may affect HRQoL and consequentially DALY losses. Similar associations of risk factors like age and prior vertebral and non-vertebral bone fractures and HRQoL were observed in the Fravo Study [25] among postmenopausal women living in the city of Valencia. Concerning the use of a drug based treatment with a SERM in the HORIZON Pivotal Fracture Trial (HORIZON-PFT) it was shown that non-SERM users had poorer general health, and on the other hand long-term drug treatment of osteoporosis has been associated with an improvement in some domains of HRQoL [33]. Although it was shown that HRQoL directly affected DALY losses in this study its mechanism is not exactly known.

A limiting factor of this study concerns the methodology used to estimate disability weights based on HRQoL data from the GINERISK study to compute DALY. Methods to estimate utility values from HRQoL data were taken from Franks et al. [23], which used a model that explained 63% of variance in EQ-5D scores. The use of different algorithms and coefficients as presented in Mortimer & Segal [34] could have affected disutility weights. Another limitation to be addressed is the cross-sectional design of the GINERISK study which provided data input for DALY calculation. The impact of co-morbidities and life style factors on HRQoL and life expectancy was not assessed and no data in the Ginerisk study was collected on the type of bone fractures. Worse HRQoL could, apart from being caused by osteoporotic bone fractures dependent on its type, also be affected by co-morbidities or lifestyle. On the contrary, specific drugs taken by participants for co-morbities could also have affected the HRQoL of postmenopausal women in the Ginerisk study, though no information was collected on other medication than osteoporosis treatment. Therefore the relationship of type of bone fracture, co-morbities and its drug treatment, and life style with DALY losses by BMD and/or age category has not been established directly and should be considered as a study limitation.

Another limitation concerns the non-availability of data on mortality for the women in the Ginerisk study. YLL in our study are an estimation of premature mortality due to osteoporosis or severe osteoporosis. To estimate the potential individual YLL of an individual participant in the Ginerisk study it is necessary to have information on the average age of death due to their condition. To give estimations for the future individual life years lost of the participants in the Ginerisk study, their mortality was based on Spanish mortality data due to osteoporosis published by the National Statistics Institute by estimating the average life years lost for each participant due to osteoporosis or severe osteoporosis according to their age at the study visit. Based on the Spanish mortality rates it was estimated that YLL due to osteoporosis or severe osteoporosis were almost neglectible compared to YLD. It needs to be stated that YLL estimated in this study concerns a future estimation and might differ based on accurate individual mortality data from the women that participated in the Ginerisk study. This data can only be collected by means of a longitudinal study over time and not in a cross-sectional study design as used for the Ginerisk study.

In total DALY were computed for 2,782 out of 4,157 Spanish postmenopausal women having an age of diagnosis of osteoporosis, a BMD (T-score) value and a HRQoL score. To examine if significant differences between women without a computed value for DALY and with a value for DALY existed, a logistic multivariate model was used. The multivariate model which examined the lack of data for computing DALY included BMD and the number of risk factors for osteoporotic bone fracture (% of right classification 85.6%) which were also tested in this study to estimate the relation of risk factors for participants with undiscounted and discounted DALY. From this multivariate model and the knowledge that these models are based on likelihood, DALY estimations in this study are valid under the assumption missing at random. It was not possible though to demonstrate that the data was missing at random, therefore the lack of the data is a limitation for this study which could have produced a lack of precision in the coefficient estimates. A possible bias could also exist in the participant selection for the GINERISK study as all women were attending outpatient gynaecology clinics.

To our knowledge this study represents the first estimation of individual DALY losses for postmenopausal women according to BMD in Spain. Mean DALY losses for the whole study population was estimated at 6.1 (95% CI 5.9-6.2). Although data on DALY losses for the total Spanish population are missing it was estimated that mean DALY losses were precise with a variation of 0.16 DALY for the bilateral 95% based on approximately 2 million women actually suffering from osteoporosis in Spain [35]. Based on this data one has to convey that osteoporosis in postmenopausal women faces an important burden of disease, particularly when a bone fracture has already occurred [34].

In the Global Burden of Disease study, osteoporosis (2.0 million DALY) accounted for 1.75% of the total DALY losses for non-communicable diseases in Europe [36,37].Other chronic diseases including asthma (1.4 million DALY), migraine (1.2 million DALY), hypertensive heart disease (1.2 million DALY), and rheumatoid arthritis (1.0 million DALY) were outranked by DALY losses due to osteoporosis. Osteoporosis has shown to constitute a considerable burden to society on a global and European level which emphasizes the health impact of this disease. Our findings have implications for future health economic analyses and policy-making related to care of osteoporosis, taking into account the full consequences of osteoporosis on the patient further than just its consequences in terms of fractures which cannot be controlled by clinicians or the healthcare system as it is traditionally defined.

## Conclusion

DALY losses were considerable amongst postmenopausal women with osteoporosis. Not having a prior bone fracture, being older, having less osteoporotic risk factors and using a SERM have been linked to less DALY losses. Risk factors affecting undiscounted and discounted DALY included alcohol consumption, rheumatoid arthritis and the use of corticosteroids.

## Abbreviations

BMD: bone mineral density
DALY: Disability-adjusted life years
DXA: Bone Densitometry
HRQoL: Health Related Quality of Life
SERM: Selective estrogen receptor modulator
YLD: Years lived with disability
YLL: Years of life lost

## References

[1] National Osteoporosis Foundation (2010). Clinician’s Guide to Prevention and Treatment of Osteoporosis.

[2] Marshall D, Johnell O, Wedel H (1996). Meta-analysis of how well measures of bone mineral density predict ocurrence of osteoporotic fractures. BMJ.

[3] Azagra R, Roca G, Encabo G, Prieto D, Aguyé A, Zwart M (1996). Prediction of absolute risk of fragility fracture at 10 years in a Spanish population: validation of the WHO FRAX ™ tool in Spain. BMC Musculoskelet Disord.

[4] Palacios S, Borrego RS, Forteza A (2005). The importance of preventive health care in post-menopausal women. Maturitas.

[5] Ferrer J, Neyro JL, Estevez A (2005). Identification of risk factors for prevention and early diagnosis of a-symptomatic post-menopausal women. Maturitas.

[6] WHO Scientific Group (2003). Prevention and Management of Osteoporosis. WHO Technical Report Series; 921.

[7] Kanis JA, Black D, Cooper C, Dargent P, Dawson-Hughes B, De Laet C (2002). A new approach to the development of assessment guidelines for osteoporosis. Osteoporos Int.

[8] Kanis JA, Melton LJ, Christiansen C, Johnston CC, Khaltaev N (1994). The diagnosis of osteoporosis. Bone Miner Res.

[9] Assessment of fracture risk and its application to screening for postmenopausal osteoporosis. Report of a WHO Study Group. WHO Technical Report Series; 843. Geneva, Switzerland: World Health Organization. 1994:5. 7941614

[10] Kanis JA, Borgstrom F, De Laet C, Johansson H, Johnell O, Jonsson B (2005). Assessment of fracture risk. Osteoporos Int.

[11] Viswanathan HN, Curtis JR, Yu J, White J, Merinar C, Stolshek BS (2012). Direct healthcare costs of osteoporosis-related fractures in managed care patients receiving pharmacological osteoporosis therapy. Appl Health Econ Health Policy.

[12] Cooper C, Campion G, Melton LJ (1992). Hip fractures in the elderly: a world-wide projection. Osteoporos Int.

[13] Gullberg B, Johnell O, Kanis JA (1997). World-wide projections for hip fracture. Osteoporos Int.

[14] Johnell O, Kanis JA (2004). An estimate of the worldwide prevalence, mortality and disability associated with hip fracture. Osteoporos Int.

[15] Wilson IB, Cleary PD (1995). Linking clinical variables with health-related quality of life. A conceptual model of patient outcomes. JAMA.

[16] Gènova-Maleras R, Catalá-López F, de Larrea-Baz NF, Álvarez-Martín E, Morant-Ginestar C (2011). The burden of premature mortality in Spain using standard expected years of life lost: a population-based study. BMC Public Health.

[17] Gardner JW, Sanborn JS (1990). Years of potential life lost (YPLL)–what does it measure?. Epidemiology.

[18] Palacios S, Neyro JL, Puertas JC, Fernandez de Cabo S (2013). Clinical profile of Spanish postmenopausal women with a diagnosis of osteoporosis and risk factors for endometrial pathology, breast cancer, and cardiovascular disease. Menopause.

[19] Fox-Rushby JA, Hanson K (2001). Calculating and presenting disability adjusted life years (DALYs) in cost-effectiveness analysis. Health Policy Plan.

[20] Murray CJL, Acharya AK (1997). Understanding DALYs. J Health Economics.

[21] Mathers CD, Vos T, Lopez AD, Salomon J, Ezzati M (2001). National Burden of Disease Studies: A Practical Guide. Global Program on Evidence for Health Policy.

[22] Instituto Nacional de Estadistíca (INE) Tablas de mortalidad de la población de España por año, sexo, edad y funciones. Esperanza de vida. [http://www.ine.es/jaxi/tabla.do?path=/t20/p319a/serie/l0/&file=01001.px&type=pcaxis&L=0] (Accessed 15 of March 2015).

[23] Franks P, Lubetkin EI, Gold MR, Tancredi DJ, Jia H (2004). Mapping the SF-12 to the EuroQol EQ-5D index in a national US sample. Med Decis Making.

[24] Sassi F (2006). Calculating QALYs, comparing QALY and DALY calculations. Health Policy Plan.

[25] Sanfélix-Genovés J, Hurtado I, Sanfélix-Gimeno G, Reig-Molla B, Peiró S (2011). Impact of osteoporosis and vertebral fractures on quality-of-life. A population-based study in Valencia, Spain (The FRAVO Study). HQLO.

[26] Cortet B, Blotman F, Debiais F, Huas D, Mercier F, Rousseaux C (2011). Management of osteoporosis and associated quality of life in post-menopausal women. BMC Musculoskelet Disord.

[27] Johnell O, Kanis JA, Oden A, Sernbo I, Redlund-Johnel I, Petterson C (2004). Mortality after Osteoporotic Fractures. Osteoporosis Int.

[28] World Health Organization (2004). The world health report: changing history.

[29] Murray CJL, Lopez AP, Murray CJL, Lopez AD (1996). Global and regional descriptive epidemiology of disability. Incidence, prevalence, health expectancies and years lived with disability. The global burden of disease: a comprehensive assessment of mortality and disability from diseases, injuries and risk factors in 1990 and projected to 2020.

[30] Murray CJL, Lopez AD, Jamison DT (1994). The global burden of disease in 1990: summary results, sensitivity analysis and future directions. Bull World Health Organ.

[31] Anand S, Hanson K (1997). Disability-Adjusted Life Years: A Critical Review. J Health Econ.

[32] Bobadilla JL, Janovsky K (1996). “Priority Setting and Cost Effectiveness”. Health Policy and Systems Development: An Agenda for Research.

[33] Sambrook PN, Silverman SL, Cauley JA, Recknor C, Olson M, Su G (2011). Health-related quality of life and treatment of postmenopausal osteoporosis: results from the HORIZON-PFT. Bone.

[34] Mortimer D, Segal L (2008). Comparing the incomparable? A systematic review of competing techniques for converting descriptive measures of health status into QALY-weights. Med Decis Making.

[35] Del Pino Montes J (2010). Epidemiología de las fracturas osteoporóticas: las fracturas vertebrales y no vertebrales. Rev Osteoporos Metab Miner.

[36] Johnell O, Kanis JA (2006). An estimate of the worldwide prevalence and disability associated with osteoporotic fractures. Osteoporos Int.

[37] International Osteoporosis Foundation (IOF). Osteoporosis is an ancient disease. [http://ec.europa.eu/health/ph_information/implement/wp/morbidity/docs/ev_20051212_co07_en.pdf] (Accessed 15 of March 2015).

